# Accuracy, inter- and intrarater reliability, and user-experience of high tibial osteotomy angle measurements for preoperative planning: manual planning PACS versus semi-automatic software programs

**DOI:** 10.1186/s40634-022-00475-x

**Published:** 2022-05-17

**Authors:** Iris E. W. G. Laven, Femke F. Schröder, Feike de Graaff, J. Christiaan Rompen, Roy A. G. Hoogeslag, Albert H. van Houten

**Affiliations:** 1 Centre for Orthopaedic Surgery and Sports Medicine, OCON, Hengelo, 7550 AM The Netherlands; 2 Techmed Centre, Faculty of Science and Technology (S&T), University of Twente, Enschede, 7522 NB The Netherlands

**Keywords:** High tibial osteotomy, Osteoarthritis, Deformity analysis, Radiographic software, Preoperative planning, accuracy, Reliability, Measurement error

## Abstract

**Purpose:**

To compare the accuracy, inter- and intrarater reliability, and user-experience of manual and semi-automatic preoperative leg-alignment measurement planning software for high tibial osteotomy (HTO).

**Methods:**

Thirty patients (31 lower limbs) who underwent a medial opening wedge HTO between 2017 and 2019 were retrospectively included. The mechanical lateral distal femur angle (mLDFA), mechanical medial proximal tibial angle (mMPTA), and planned correction angle were measured on preoperative long-leg full weight-bearing radiographs utilising PACS Jivex Review® v5.2 manual and TraumaCad® v2.4 semi-automatic planning software. Independent measurements were performed by four raters. Two raters repeated the measurements. Accuracy in the standard error of measurement (SEM), inter- and intrarater reliability, and user-experience were analysed. Additionally, measurements errors of more than 3° were remeasured and reanalysed.

**Results:**

The SEMs of all measured varus malalignment angles and planned correction angle were within 0.8° of accuracy for both software programs. Measurements utilising the manual software demonstrated moderate interrater intraclass correlation coefficient (ICC)-values for the mLDFA and mMPTA, and an excellent interrater ICC-value for the correction angle (0.810, 0.779, and 0.981, respectively). Measurements utilising the semi-automatic software indicated excellent interrater ICC-values for the mLDFA, mMPTA, and correction angle (0.980, 0.909, and 0.989, respectively). The intrarater reliability varied substantially per angle, presenting excellent intrarater agreements by both raters (ICC >  0.900) for the correction angle in each software program as well as poor-to-excellent ICC-values for the mLDFA (0.282–0.951 and 0.316–0.926) and mMPTA (0.893–0.934 and 0.594–0.941) in both the manual planning and semi-automatic software. Regarding user-experience, semi-automatic software was preferred by two raters, while the other two raters had no distinctive preference. After remeasurement of five outliers, excellent interrater ICC-values were found for the mLDFA (0.913) and mMPTA (0.957).

**Conclusions:**

Semi-automatic software outperforms the manual software when user-experience and outliers are considered. However, both software programs provide similar performance after remeasurement of the human-related erroneous outliers. For clinical practice, both programs can be utilised for HTO planning.

**Level of evidence:**

Diagnostic study, Level III.

**Supplementary Information:**

The online version contains supplementary material available at 10.1186/s40634-022-00475-x.

## Introduction

High tibial osteotomy (HTO) to correct varus malalignment of the leg is an effective joint-preserving surgical technique to treat medial compartment knee osteoarthritis (OA) in young, active patients [9, 15, 23]. By realigning the mechanical weight-bearing leg axis, the weight-bearing forces are redistributed, decreasing the forces on the medial knee compartment and therefore preventing progression of medial OA [7, 18, 34]. Preoperatively, a malalignment analysis and measurement of the planned correction angle are determined by knee joint angle measurements on long-leg full weight-bearing radiograph [24]. It is important to measure these angles accurately in order to plan and achieve an optimal deformity angle of correction during surgery, since measurement error affects accuracy [20, 21, 28, 33].

Inaccurate preoperative osteotomy planning can be caused by known insufficiencies including inconsistent patient positioning and system setup during radiograph acquisition [2, 3, 5, 22, 33], and lack of awareness of unintentional alteration of adjacent joints [1, 12]. Other than the aforementioned insufficiencies, the accuracy of the preoperative angle measurements and planning of the correction angle utilising software programs is important to take into account.

Various manual and semi-automatic software programs can be utilised to perform the deformity analysis and determine the planned correction angle. Research to date has provided excellent rater agreements for both types of software programs with inter-, and intrarater intraclass correlation coefficients (ICCs) between 0.841 and 0.998 (Table 1) [6, 20, 21, 25–27, 33]. Apart from the ICC, there are other possibilities to evaluate accuracy in digital planning, including the measurement error. Because of the huge influence of measurement errors on complication rates and outcome, the measurement error should be considered if accuracy of digital planning will be assessed. Nevertheless, evidence regarding measurement errors for lower limb deformity angles is scarce. To the best of the authors’ knowledge, only Nerhus et al. (2017) reported measurement errors for the femoral and tibial malalignment angles with values up to 2.1° [21]. Measurement errors of 2° imply a substantial risk of unintended over- or underestimation of the size of the varus malalignment, since under- or overcorrection of 1° in the coronal plane can result in progression of medial OA [28, 32]. Additionally, no previous study has investigated the measurement error of the planned correction angle while specifically utilising manual or semi-automatic planning software programs.

**Table 1:** Summary of literature reporting the rater agreements of the deformity and planned correction angle measurements [6, 20, 21, 25–27, 33]

Literature	N	k	Materials	mLDFA	mMPTA	Planned correction angle
Elson et al. (2013) [6]	24	3	PACS Viewer (manual)^a^	NR	NR	ICC-1: (0.980–0.986) ICC-2: (0.965–0.985)
Munier et al. (2017) [20]	10	2	Centricity software® (GE Healthcare)	NR	ICC-1: 0.980 ICC-2: 0.920	NR
Nerhus et al. (2017) [21]	50	2	MediCad v2.24 module osteotomy	ICC-1: 0.91 | CR/SRD: 1.9° ICC-2: 0.89 | CR/SRD: 2.1°	ICC-1: 0.91 | CR/SRD: 1.9° ICC-2: 0.89 | CR/SRD: 2.1°	NR
Schröter et al. (2012) [25]	81	3	PreOPlan®^a^	ICC-1: 0.841 (0.780–0.889)	ICC-1: 0.974 (0.963–0.983)	ICC-1: 0.993 (0.990–0.995)
			MediCad®^a^	ICC-1: 0.947 (0.925–0.964)	ICC-1: 0.974 (0.961–0.983)	ICC-1: 0.995 (0.992–0.996)
Segev et al. (2010) [26]	10	5	TraumaCad®	ICC-1: 0.630–0.950	ICC-1: 0.690–0.810	NR
Sled et al. (2011) [27]	105	7	AutoCad® manual^a^	ICC-1: 0.990 (0.983–0.995)	ICC-1: 0.906 (0.843–0.948)	NR
	200	3	AutoCad semi-automatic^a^	ICC-1: 0.960 (0.953–1) ICC-2: 0.966 (0.961–1)	ICC-1: 0.947 (0.937–1) ICC-2: 0.964 (0.959–1)	NR
Yazdanpanah et al. (2017) [33]	108	3	Software Medview Meddiag® v3.0.4	Inter: > 0.99 Intra: > 0.99	Inter: 0.92 Intra: > 0.99	NR

^a^Mean (Confidence interval 95%)

*CR/SRD* coefficient of repeatability/smallest real difference, *ICC*-1 interrater intraclass correlation coefficient, *ICC*-2 intrarater intraclass correlation coefficient, *k* number of raters, *mLDFA* mechanical lateral distal femoral angle, *mMPTA* mechanical medial proximal tibial angle, *N* number of legs measured, *NR* not reported

Consequently, as current literature does not report all required performance metrics of both types of planning software programs, this study compares the accuracy of manual and semi-automatic software programs in measurement error size, inter- and intrarater reliability, and user-experience for deformity analysis as well as measurements of planned correction angle in HTO procedures. The user-experience of these software programs for analysing deformity and measuring the planned correction angle has not been published previously. The authors hypothesise that the semi-automatic planning software measurements produce lower measurement errors, higher inter- and intrarater reliability, and increased user-experience.

## Methods

This study was developed in accordance with the Guidelines for Reporting Reliability and Agreement Studies (GRASS) [14].

### Patients

**Fig. 1:**
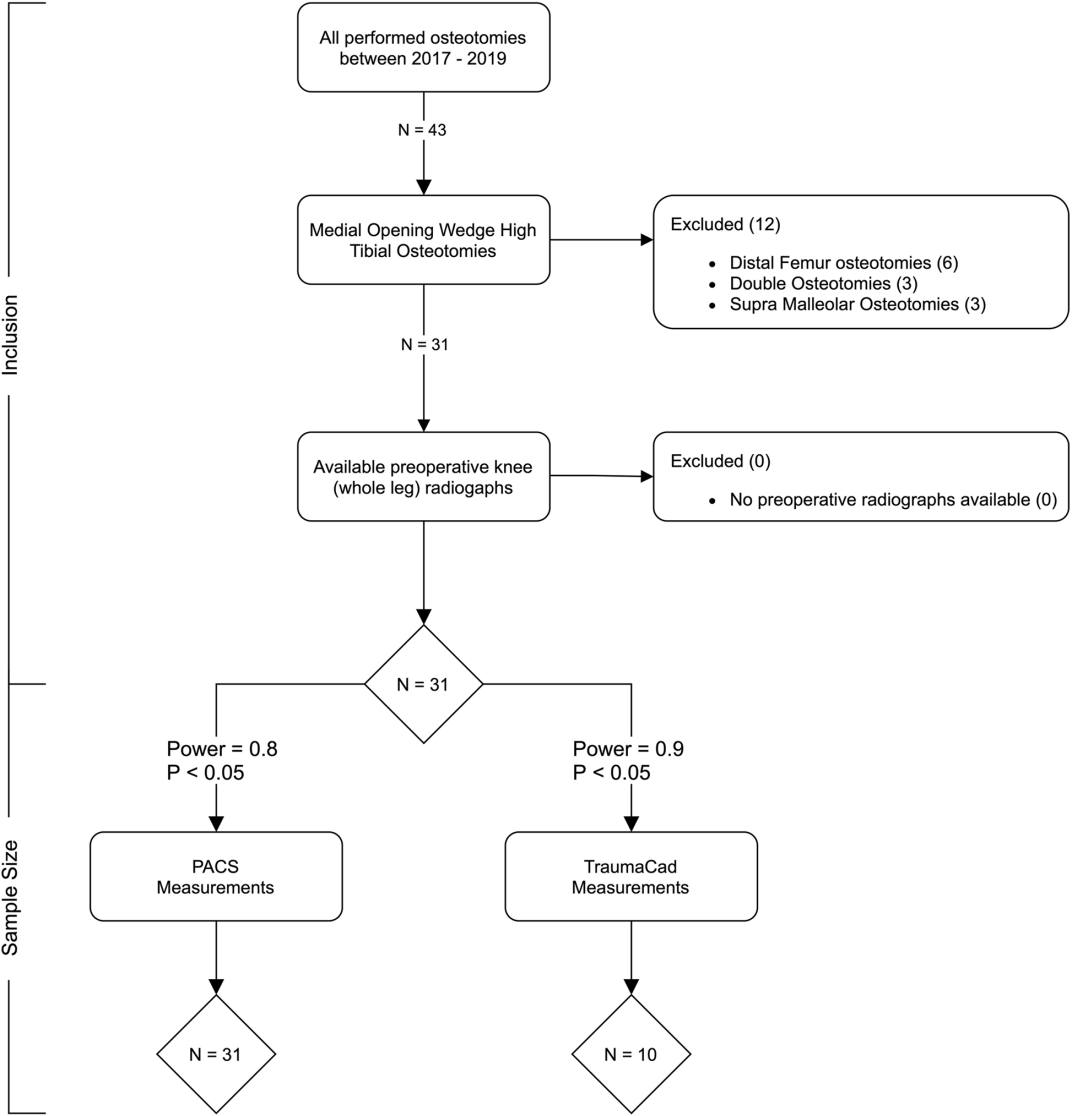
Flowchart of patient selection

Patients who underwent a medial opening wedge HTO between 2017 and 2019 were potentially eligible for inclusion in the present study. Patients who underwent an additional distal femur osteotomy or supramalleolar ankle osteotomy were excluded (Fig. 1) [29]. Thirty-one osteotomies (15 right-knee and 16 left-knee procedures) in 30 patients (26 males and 4 females) with a mean age of 45 (± 12) years, BMI of 26.3 (± 3.3) kg/m2, Kellgren and Lawrence OA grade [11] of 2 (min. 1 – max. 3), and a mean varus deformity of 5.4° (± 2.3°) were included.

### Data acquisition

Long-leg full weight-bearing radiographs were acquired preoperatively utilising a Siemens Healthineers’ Thorax/Multix FD SD® (Siemens Healthcare GmbH, Germany) digital radiography system with a universal grid. The X-ray beam was centred at the knee and was positioned at a fixed distance of 300 cm from the patient. Settings of 77–90 kV were utilised, depending on the tissue characteristics and length of the lower extremity. Patients were required to stand, bearing full weight on both legs with 0° of leg extension in the anterior-posterior direction with the patella centralised over the distal femoral condyles. Anterior-posterior and lateral radiographs of the affected knee were acquired with the same X-ray system, utilising a 60 kV setting and a universal grid positioned approximately 120 cm from the source to the detector.

### Measurements

For malalignment analysis, the mechanical lateral distal femur angle (mLDFA) and the mechanical medial proximal tibial angle (mMPTA) were measured. The planned correction angle was measured utilising Miniaci’s method [6, 10, 19].

### Manual planning software angle measurements

**Fig. 2:**
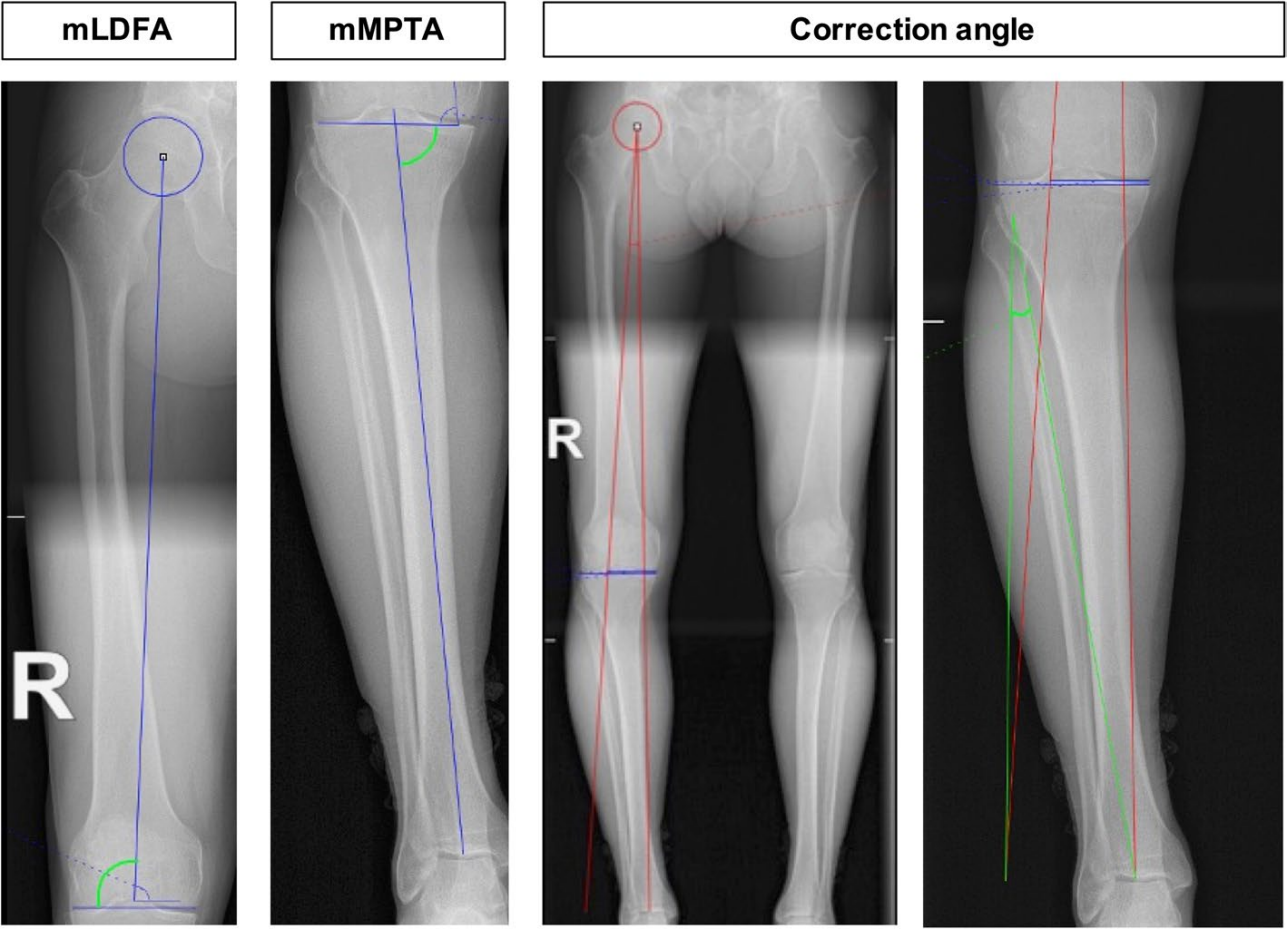
Deformity analysis and planned correction angle measurements acquired utilising the manual planning PACS software Jivex®. The angles include the measurements of the mLDFA, mMPTA, correction angle, and the correction angle. The green angle presents the corresponding measurement value. mLDFA = mechanical lateral distal femoral angle; mMPTA = mechanical medial proximal tibial angle

Basic tools are provided by the manual PACS software Jivex® v5.2 (VISUS Technology Transfer GmbH, Germany), which include circle, ruler, and straight-line tools. An overview of the aforementioned angle measurements is included in Fig. 2. To measure the mLDFA, the centre of the femoral head was determined utilising the electronic option ‘circle with centre-point’. Subsequently, the option ‘open angle measurement’ was selected, and one straight line was drawn from the previously selected femoral centre-point to the centre of the knee; a second straight line was drawn between the two most distal (convex) points of the femoral condyles. For the mMPTA measurement, only the ‘open angle measurement’ was required, so a straight line was drawn between the most convex points of the medial and lateral tibial plateaus, and a second straight line was drawn between the centre of the knee and centre of the talar dome, which represents the mechanical tibial axis. Finally, the correction angle was calculated based on Miniaci’s measurement method [19], as explained by Elson et al. [6]: first, the position of Fujisawa’s point, based on a weight-bearing axis of 62.5% through the tibial plateau, was calculated utilising the ‘line-relation measurement’ option. Second, the option ‘open angle measurement’ was utilised, and a straight line was drawn, representing the Mikulicz line (preoperative weight-bearing line), as was a straight line from the centre of the hip through Fujisawa’s point to the height of the talar dome. Finally, the option ‘open angle measurement’ was utilised to calculate the correction angle between the two straight lines intersecting at the hinge point. The software program reported the angle measurements to one decimal.

### Semi-automatic software angle measurements

**Fig. 3:**
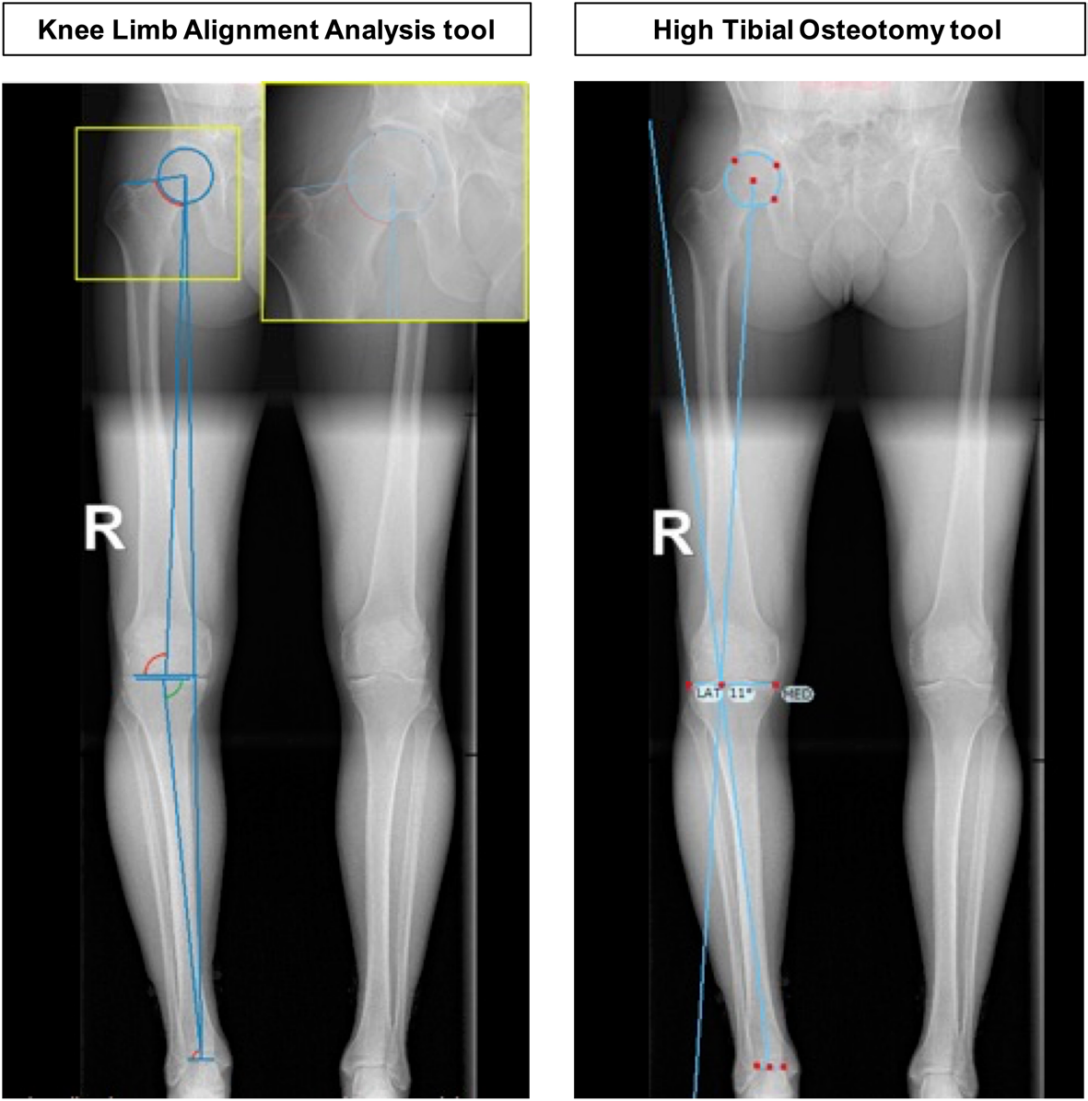
Deformity analysis and planned correction angle measurements utilising the semi-automatic software tools in TraumaCad®. The Knee Limb Alignment Analysis Tool was utilised to determine the mLDFA and mMPTA based on the centre of the hip, knee, and ankle (left). The High Tibial Osteotomy Tool was utilised to determine the size of the correction angle required to restore the mechanical leg axis (right). mLDFA = mechanical lateral distal femoral angle; mMPTA = mechanical medial proximal tibial angle

TraumaCad v2.4 (VoyantHealth, BrainLab Company, Israel) includes two semi-automatic tools to measure the deformity angles and planned correction angle. The ‘Knee Limb Alignment Analysis tool’ was utilised to measure the mLDFA and mMPTA. In this program, the user selects the centre of the hip based on a three-point circle method: the top of the greater trochanter, the most distal points of the medial and lateral femoral condyles, the most distal points of the medial and lateral tibial plateau, and the most medial and lateral points of the tibial plafond. Then, the ‘High Tibial Osteotomy tool’ was utilised to calculate the HTO correction angle. Figure 3 presents an overview of the semi-automatic software’s joint angle measurements. The software program provides the results in integers.

### Rating process

Three orthopaedic knee surgeons (R1, R2, and R3) and the primary investigator (R4), who was trained by the orthopaedic knee surgeons, independently performed measurements utilising the two software programs. Uniformity of the measurement methods for each radiological parameter was ensured by a single training session prior to commencing the measurements. R1 and R4 performed the measurements again after approximately 6 weeks with a different presentation order of the cases. Because of technical limitations, it was not possible to blind the raters from the subjects’ patient identification numbers.

### User-experience

The user-experience of the software programs was investigated utilising the Post Study System Usability Questionnaire (PSSUQ), which is widely employed to assess user-experience and offers excellent reliability [16, 17]. Its 19 questions must be answered on a 7-point scale, where 1 indicates ‘Strongly Agree’ and 7 indicates ‘Strongly Disagree’. These questions are included in Additional File 1. The PSSUQ’s output includes an average overall score, average System Usefulness (SYSUSE), average Information Quality (INFOQUAL) and an average Interface Quality (INTERQUAL) scores [17].

### Statistical analysis

The required sample size for a reliability analysis of the continuous angle measurements was determined by the predicted ICC of the interrater reliability using Temel et al.’s method [29]. For the manual and semi-automatic software measurements, ICC-values around 0.800 and 0.900 were predicted, respectively. With a power of 0.8 and a significance level of *P* < 0.05, a minimum of 31 patients for the manual software and 10 for the semi-automatic software were required.

The accuracy of each angle was determined via the standard error of measurement (SEM), which was derived from the formula SEM = SD * sqrt(1 – ICC) [30, 31], and includes the standard deviation (SD) of the measurement error and the ICC. A measurement error was defined as the difference between the individual rater’s measurement and the overall rater average of the angle measurement on the same long-leg full weight-bearing radiograph.

Interrater reliability analyses were performed utilising a two-way random effects model with the absolute agreement of the average measurements of all raters. A two-way random effects model with the absolute agreement of the single measurements was utilised to determine the intrarater reliability. ICC-values were reported with corresponding 95% confidence intervals (CI). Values between 0–0.500, 0.500–0.750, 0.750–0.900, and 0.900–1.000 indicate poor, moderate, good and excellent reliability, respectively [13].

Additionally, the similarity between the software programs for each HTO angle was analysed via a paired t-test (normal distribution) or Wilcoxon signed-rank test (non-normal distribution) to determine any software-related significant mean differences. Normality of the data was assessed utilising the Shapiro-Wilk test, considering non-normality at *P <* 0.05. Statistical analyses were conducted with SPSS version 26.0 (IBM SPSS Statistics, Inc.; Armonk, NY, USA).

### Additional analysis

An individual rater's measurement that differed more than 3° from the overall rater average on the same long-leg full weight-bearing radiograph was considered an outlier, and was remeasured by the corresponding rater. Outliers were analysed independently and possible human errors were identified. Subsequently, remeasurement of the outliers was performed to either confirm or reject the identified cause (i.e., human error).

## Results

**Fig. 4:**
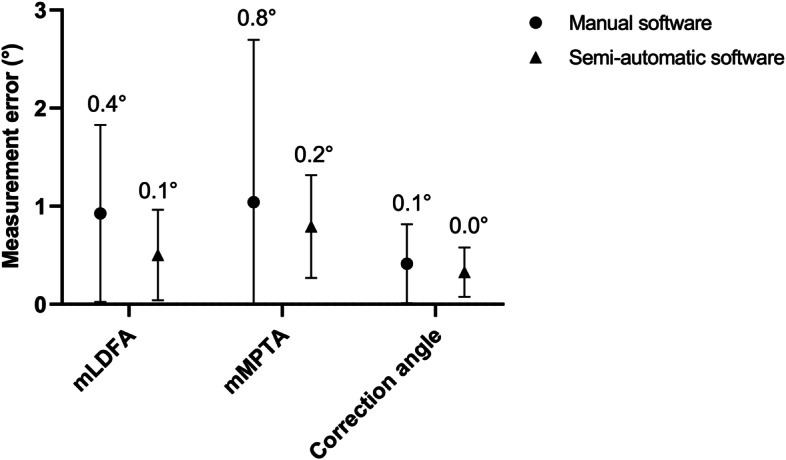
Measurement errors. Mean and standard deviation of the measurement errors of all raters per angle per software. The corresponding SEM was displayed above each error bar. SEM = standard error of measurements

Figure 4 presents the absolute measurement errors of all raters of the mLDFA, mMPTA, and correction angle per software program. Accuracy was calculated via the SEM and was found to be within 0.8° for all HTO angles (min. 0.0 – max. 0.8°) for both software programs.

**Fig. 5:**
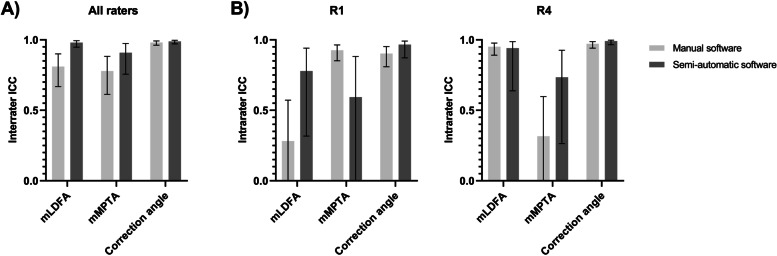
Interrater and intrarater analyses. ICC-values of the mLDFA, mMPTA and correction angle of A) the interrater agreement of all raters, and B) the intrarater agreement for R1 and R4. ICC = intraclass correlation coefficient; mLDFA = mechanical lateral distal femoral angle; mMPTA = mechanical medial proximal tibial angle; R = rater

As shown in Fig. 5A, manual software measurements provided moderate interrater reliability for the mLDFA (0.810; 95%-CI 0.668–0.900) and mMPTA (0.779; 95%-CI 0.613–0.883), and excellent interrater reliability for the planned correction angle (0.981; 95%-CI 0.962–0.992). For the semi-automatic software, excellent interrater reliability scores were found for all angles with ICC-values of 0.980 (95%-CI 0.948–0.994, 0.909 (95%-CI 0.756–0.974) and 0.989 (95%-CI 0.971–0.997) for the mLDFA, mMPTA and the correction angle respectively. The intrarater reliability varied per software (Fig. 5B), per rater and per angle, except for the correction angle, which demonstrated excellent agreement (ICC >  0.903) in both software programs. Poor reproducibility of the mLDFA measurements (0.282; 95%-CI -0.065–0.572) was found for R1, whereas R4 had poor reproducibility values for the mMPTA measurements (0.316; 95%-CI -0.030–0.597).

Similarity analyses revealed no software-related significant mean differences for the mLDFA and mMPTA (*P* = 0.127 and *P* = 0.782, respectively). There was a significant difference of − 0.5° (*P* < 0.001) between the programs for the correction angle.

Regarding user-experience, the semi-automatic software was preferred over the manual software by two raters (R3 and R4) based on the SYSUSE and INFOQUAL (Table 2). R1 and R2 had no distinctive preference regarding software programs.

**Table 2:** Overview of the user-experience scores measured utilising the PSSUQ

	R1		R2		R3		R4	
	Manual	Semi-automatic	Manual	Semi-automatic	Manual	Semi-automatic	Manual	Semi-automatic
SYSUSE	1.8	2.0	1.8	2.0	2.7	1.7	3.7	1.7
INFOQUAL	2.0	2.0	2.3	2.3	3.0	2.0	4.7	2.4
INTERQUAL	2.0	2.0	2.0	2.0	1.3	2.0	2.0	1.7
Overall	1.9	2.0	2.0	2.1	2.4	1.8	3.6	1.9

*INFOQUAL* information quality, *INTERQUAL* interface quality, *SYSUSE* system usefulness

Seven-point scale: 1 = ‘Strongly Agree’; 7 = ‘Strongly Disagree’

### Additional analysis

No outliers were identified for the semi-automatic software angle measurements. Five manual software measurements, three mMPTA and two mLDFA measurements, had a measurement error of more than 3° compared to the overall rater average of the angle measurement on the same long-leg full weight-bearing radiograph (Table 3) and were therefore remeasured by R1 and R4. Analyses of the remeasurements of the mMPTA and mLDFA showed SEM values of 0.3° and 0.2°, as well as interrater ICC-values of 0.913 (95%-CI 0.820–0.958) and 0.957 (95%-CI 0.917–0.978), respectively. The intrarater ICC value for the mLDFA by R1 increased to moderate agreement (0.514; 95%-CI 0.210–0.729), while the intrarater ICC value of the mMPTA by R4 increased to excellent (0.934; 95%-CI 0.869–0.968).

**Table 3:** Measurement outliers of more than 3° compared to the overall rater average of the angle measurement on the same long-leg full weight-bearing radiograph

Outlier	Software program	Angle	Angle measurement	Difference from overall rater average	Rater
1	Manual software	mLDFA	86.2°	- 3.6°	R1
2	Manual software	mLDFA	83.0°	- 3.8°	R1
3	Manual software	mLDFA	94.5°	+ 6.8°	R1
4	Manual software	mMPTA	98.2°	+ 13.2°	R4
5	Manual software	mMPTA	95.9°	+ 9.7°	R4

## Discussion

The most important finding of the present study is that manual planning has a higher risk of outliers in deformity angle measurements. After remeasurement of the outliers that were related to human errors, both software programs gave similar results in terms of accuracy (< 0.3°) and inter- and intrarater reliability for HTO angle measurements. Regarding user-experience, two out of four raters preferred the semi-automatic software, whereas the other two raters had no distinctive preference. Based on these results, the hypothesis that the performance of semi-automatic software would be superior to manual software programs for HTO planning cannot be rejected nor be confirmed completely.

The accuracy of the preoperative HTO angle measurements is important for optimal deformity correction, which ensures the long-term success of an HTO [20, 21, 28, 33]. However, the study by Nerhus et al. (2017) and the current study are the only reports that quantify the measurement error [21]. Nerhus et al. (2017) report the correlation of repeatability (CR) scores for the mLDFA and mMPTA utilising semi-automatic medical planning software (mediCAD v2.24 osteotomy module); these scores can be converted to acquire the SEM (CR = SEM*2.77). The present study reveals lower measurement errors than Nerhus et al. (2017) for the mLDFA (0.4° vs. 0.7°). For the mMPTA measurements, a lower error was found after remeasurement of the outliers which were related to typographical errors (0.2° vs. 0.7°) [21]. These small differences in accuracy (less than 1°) may not be clinically relevant, since under- or overcorrection of 1° or more has been related to the progression of OA [28, 32]. This insinuates that, in clinical practice, both manual and semi-automatic software programs provide accurate preoperative HTO planning for varus malalignment. Nonetheless, it should be noted that several outliers were identified for the manual software angle measurements.

Regarding interrater reliability, the results herein correspond with the interrater reliabilities of manual and semi-automatic software programs reported in other studies (Table 1). However, the authors found moderate interrater ICC-values for the mLDFA as measured with the manual software. In-depth analysis of the outliers revealed that the moderate interrater ICC-values for the mLDFA that were obtained utilising the manual software seemed to be related to the three incorrect mLDFA measurements by R1. These incorrect measurements probably occurred due to the colour blindness of R1 as the level of colour contrast between the measurement lines and the long-leg full weight-bearing radiograph in the user interface of the manual software program is low. A post-hoc analysis excluding the data of R1, demonstrated a remarkable change in mLDFA measurements utilising the manual software from a mean ICC-value of 0.810 to a mean ICC-value of 0.918. However, this difference is most likely caused by absence of the outliers which were measured by R1, as the remeasured outlier data including the data of R1 shows a similar ICC-value of 0.913.

The reported intrarater reliabilities in this study were moderate for the mLDFA and mMPTA measurements that were utilised with the semi-automatic software, whereas previous literature reveals good-to-excellent intrarater reliability scores (Table 1) [21, 25, 27, 33]. The lower intrarater reliability scores may be related to the smaller sample size measured for the semi-automatic software measurements, and the precision of the semi-automatic software measurements (that were rounded up to integers instead of decimals).

The user-experience was scored utilising the PSSUQ [16, 17], which scores the overall functionality based on the software’s usefulness in its interface, system usability, and information quality. Two raters preferred the semiautomatic software, predominantly because of the SYSUSE. These raters were younger and less experienced (1 and 5 years) with the manual HTO angle measurements compared to the other raters (> 10 years). In spite of the lack of experience with TraumaCad's semi-automatic HTO angles measurements by the raters, these values indicate that the program was user-friendly and useful after only a few HTO angle measurements (*N* = 10).

The outliers of R1 might have been caused by the potential influence of colour-blindness. The outliers of R4 were most likely related to misinterpretation of the measured angle. In case the outliers remained unnoticed, these could potentially have led to a false indication of the size and the origin of the deformity. One of the three mLDFA outliers was higher than 90°, indicating the deformity would have been located in the femur and may have led to a double osteotomy. With regard to the mMPTA measurements, the lines were made correctly, but the right interpretation of the measured angle was not made. Notwithstanding, the correction angles did not experience poor to moderate reliability and reproducibility measurements, but had excellent reliability and producibility, suggesting the orthopaedic surgeons would have noticed these incorrect measurements during the simulation of the correction.

This study has several strengths and limitations. The main strength is that it is one of the first studies in which accuracy measurements were conducted and the user-experience of manual and semi-automatic software programs was investigated regarding HTO planning. However, this study was limited by software-related restrictions. First, it was not possible to anonymise patient data, which may have allowed raters to recall their doctor-patient interactions. Second, the low-contrast ratio of the manual software program’s user interface potentially resulted in misinterpreted angle measurements by the rater with colour blindness (R1). Furthermore, there was a significant difference in similarity for the correction angle between the software programs. Nonetheless, this difference was lower than 1° and may therefore be clinically irrelevant [28, 32]. Additionally, the results of the semi-automatic software could potentially have been biased by the lower number of measurements compared to the manual software, as well as by a possible learning curve since not all raters had used the semi-automatic software before. These biases may have led to an underestimation of the semi-automatic performance.

In addition to the PSSUQ items to score user-experience, it would be interesting to include the measurement time for all HTO angles. Semi-automatic tools are potentially less labour-intensive compared to manual angle measurements. However, TraumaCad had not been utilised prior to this study, hence the measurement time may have been biased by a learning curve.

For optimal osteotomy planning using long-leg full weight-bearing radiographs, guidelines for system setup and an easy-to-implement protocol for patient positioning should be developed to minimize measurement errors during acquisition of radiographs [2, 3, 5, 22, 33]. Additionally, orthopaedic surgeons have to bear in mind that the alignment of adjacent joints may be unintentionally altered as a result of a planned osteotomy which could lead to undesirable effects, such as secondary OA of the ankle [1, 12]. These unforeseen changes in the alignment of adjacent joints may be limited by using a semi-automatic software program which automatically displays all frontal lower limb angles during simulation of the correction, simultaneously shows (un)wanted angle changes and provides recommendations for alterations [4, 8].

## Conclusion

This study investigated the accuracy, inter- and intrarater reliability, and user-experience of HTO angle measurements acquired utilising manual and semi-automatic software. The semi-automatic software outperforms the manual software when user-experience and measurement outliers are considered. However, after remeasurement of the human-related erroneous outliers, no significant differences were found between the software programs in accuracy, or inter- and intrarater reliability, indicating a low risk of unintended under- or overcorrection of the planned varus malalignment correction. Both programs can be utilised for HTO malalignment analysis in clinical setting.

## Supplementary Information

**Additional file 1.** Post Study System Usability Questionnaire (PSSUQ). This table presents the PSSUQ questions by category of usability.

## Abbreviations

CI: Confidence intervals
GRASS: Guidelines for Reporting Reliability and Agreement Studies
HTO: High tibial osteotomy
ICC: Intraclass correlation coefficients
INFOQUAL: Information Quality
INTERQUAL: Interface Quality
mLDFA: Mechanical lateral distal femur angle
mMPTA: Mechanical medial proximal tibial angle
OA: Osteo-arthritis
PSSUQ: Post Study System Usability Questionnaire
SD: Standard deviation
SEM: Standard errors of measurements
SYSUSE: System Usefulness
