# Supplementary material for: Accuracy, inter- and intrarater reliability, and user-experience of high tibial osteotomy angle measurements for preoperative planning: manual planning PACS versus semi-automatic software programs
Source: J Exp Orthop. 2022 May 17;9:44. doi: 10.1186/s40634-022-00475-x (PMC9114281; doi:10.1186/s40634-022-00475-x)
Supplement: Supplementary file 1 — Additional file 1. Post Study System Usability Questionnaire (PSSUQ). This table presents the PSSUQ questions by category of usability. [file 40634_2022_475_MOESM1_ESM.pdf]

Additional file 1: Post Study System Usability Questionnaire (PSSUQ).

	System Usefulness (SYSUSE)	Points [a]
1.	Overall, I am satisfied with how easy it is to use this system.	
2.	It was simple to use this system.	
3.	I was able to complete the tasks and scenarios quickly using this system.	
4.	I felt comfortable using this system.	
5.	It was easy to learn to use this system.	
6.	I believe I could become productive quickly using this system.	

	Information Quality (INFOQUAL)	Points [a]
7.	The system gave error messages that clearly told me how to fix problems.	
8.	Whenever I made a mistake using the system, I could recover easily and quickly.	
9.	The information (such as online help, on-screen messages, and other documentation) provided with this system was clear.	
10.	It was easy to find the information I needed.	
11.	The information was effective in helping me complete the tasks and scenarios.	
12.	The system gave error messages that clearly told me how to fix problems.	

	Interface Quality (INTERQUAL)	Points [a]
13.	The interface of this system was pleasant.	
14.	I liked using the interface of this system.	
15.	This system has all the functions and capabilities I expect it to have.	

	Overall	Points [a]
16.	Overall, I am satisfied with this system.	

[a] Agreement 7 points scale | 1 = Strongly Agree; 7 = Strongly Disagree; N.A. = Not Applicable

Article Title: Accuracy, inter- and intrarater reliability, and user-experience of high tibial osteotomy angle measurements for preoperative planning: manual planning PACS versus semi-automatic TraumaCad software programs

Journal Name: Journal of Experimental Orthopaedics (JEO)

Authors: Iris E.W.G. Laven, MSc; Femke F. Schröder, PhD; Feike de Graaff, PhD; J. Christiaan Rompen, MD; Roy A.G. Hoogeslag, MD; Albert H. van Houten, MD, PhD

Corresponding author: Feike de Graaff, Research coordinator; Centre for Orthopaedic Surgery and Sports Medicine, OCON, Geerdinksweg 141 postbus 546, 7550, AM, Hengelo, The Netherlands, Email: f.dgraaff@ocon.nl; Phone: +31 88 708 33 70
